# Left auditory cortex gamma synchronization and auditory hallucination symptoms in schizophrenia

**DOI:** 10.1186/1471-2202-10-85

**Published:** 2009-07-20

**Authors:** Kevin M Spencer, Margaret A Niznikiewicz, Paul G Nestor, Martha E Shenton, Robert W McCarley

**Affiliations:** 1 Research Service, Veterans Affairs Boston Healthcare System and Department of Psychiatry, Harvard Medical School, Research 151C, 150 S. Huntington Ave, Boston, MA 02130, USA; 2 Research Service, Veterans Affairs Boston Healthcare System and Department of Psychiatry, Harvard Medical School, Psychiatry 116A, 940 Belmont St, Brockton, MA 02301, USA; 3 Mental Health Service, Veterans Affairs Boston Healthcare System and Department of Psychiatry, Harvard Medical School, Psychiatry 116A, 940 Belmont St, Brockton, MA 02301, USA; 4 Department of Psychology, University of Massachusetts, Boston, 100 Morrissey Blvd, Boston, MA 02125, USA; 5 Department of Psychiatry, Brigham and Women's Hospital and Harvard Medical School, Psychiatry Neuroimaging Laboratory, 1249 Boylston St, Boston, MA 02215, USA

## Abstract

**Background:**

Oscillatory electroencephalogram (EEG) abnormalities may reflect neural circuit dysfunction in neuropsychiatric disorders. Previously we have found positive correlations between the phase synchronization of beta and gamma oscillations and hallucination symptoms in schizophrenia patients. These findings suggest that the propensity for hallucinations is associated with an increased tendency for neural circuits in sensory cortex to enter states of oscillatory synchrony. Here we tested this hypothesis by examining whether the 40 Hz auditory steady-state response (ASSR) generated in the left primary auditory cortex is positively correlated with auditory hallucination symptoms in schizophrenia. We also examined whether the 40 Hz ASSR deficit in schizophrenia was associated with cross-frequency interactions.

Sixteen healthy control subjects (HC) and 18 chronic schizophrenia patients (SZ) listened to 40 Hz binaural click trains. The EEG was recorded from 60 electrodes and average-referenced offline. A 5-dipole model was fit from the HC grand average ASSR, with 2 pairs of superior temporal dipoles and a deep midline dipole. Time-frequency decomposition was performed on the scalp EEG and source data.

**Results:**

Phase locking factor (PLF) and evoked power were reduced in SZ at fronto-central electrodes, replicating prior findings. PLF was reduced in SZ for non-homologous right and left hemisphere sources. Left hemisphere source PLF in SZ was positively correlated with auditory hallucination symptoms, and was modulated by delta phase. Furthermore, the correlations between source evoked power and PLF found in HC was reduced in SZ for the LH sources.

**Conclusion:**

These findings suggest that differential neural circuit abnormalities may be present in the left and right auditory cortices in schizophrenia. In addition, they provide further support for the hypothesis that hallucinations are related to cortical hyperexcitability, which is manifested by an increased propensity for high-frequency synchronization in modality-specific cortical areas.

## Background

Hallucinations – perceptions without a basis in the physical environment – are a hallmark symptom of schizophrenia. Since the precise synchronization of neural activity in the γ band (30–100 Hz) of the electroencephalogram (EEG) may underlie the representation of perceptions [1], γ oscillation abnormalities could be related to hallucinations in schizophrenia. Previously we have reported that the history of visual hallucination symptoms in chronic schizophrenia patients was positively correlated with the degree of phase locking of a β oscillation recorded over visual cortex [2]. In another study, we found that the overall hallucination history of first-episode schizophrenia patients was positively correlated with the phase locking of the 40 Hz harmonic of the 20 Hz auditory steady-state response (ASSR) recorded over fronto-central electrodes [3]. The positive direction of the correlation in both studies was unexpected, as most reports of oscillatory activity in schizophrenia patients have observed reductions in power and/or phase synchronization relative to healthy individuals (e.g., [4-7]).

Taken together, our previous two observations suggest that a greater propensity for hallucinations in schizophrenia is associated with increased phase locking of high-frequency oscillations in the scalp-recorded EEG [8], even when patients are not actively hallucinating. While we did not utilize a symptom rating scale with items for modality-specific hallucinations in the above-mentioned ASSR study, the overall hallucination scale likely reflected primarily auditory hallucinations. Since the cortical generators of the ASSR are located in the primary auditory cortex (e.g., [9,10]), and the visual β oscillation was observed over occipital cortex, oscillations demonstrating positive correlations between phase synchronization and hallucination symptoms may originate in sensory cortical areas associated with the modality of the hallucinations. Consistent with this hypothesis, Baldeweg and colleagues reported that a psychiatric patient experiencing somatic hallucinations evinced γ oscillations over sensorimotor cortex [11], and Ropohl et al. [12] found abnormally large β oscillations localized to the left auditory cortex in a schizophrenia patient with treatment-resistant auditory hallucinations.

There is a good deal of evidence implicating the language-related areas of the left cerebral hemisphere (LH) in auditory hallucinations in schizophrenia (e.g., [13,14]). To date there have also been reports that the structure [15] and activity [16-19] of the left primary auditory cortex (Heschl's gyrus) is related to auditory hallucinations as well. Since γ oscillations may reflect cortical circuitry abnormalities in schizophrenia (reviewed in [20]), which have been observed in the primary auditory cortex [21,22], and the phase synchronization and power of γ-band ASSRs are reduced in schizophrenia [3,6,23-26], here we used the 40 Hz ASSR to investigate whether the propensity for auditory hallucinations might be associated with increased γ synchronization in the left primary auditory cortex. The activity of ASSR sources in the primary auditory cortex of chronic schizophrenia patients and healthy control subjects was measured using time-frequency decomposition in conjunction with dipole source localization. In addition, we examined whether the ASSR deficit might involve cross-frequency interactions, as a recent study demonstrated that oscillatory activity in the auditory cortex of macaques is organized hierarchically, with higher-frequency oscillations being modulated by the phase of lower-frequency oscillations [27].

## Results

### Scalp EEG data

**Figure 1:**
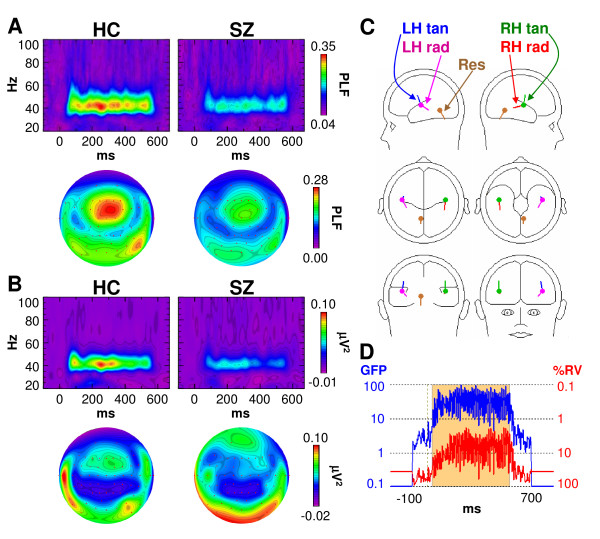
**Analyses of scalp EEG data**. **(A) **Time-frequency and topographic maps of phase locking factor (PLF) for healthy controls (HC) and schizophrenia patients (SZ). The time-frequency maps show responses at electrode Fz. Topographic maps are oriented so that the top of the map is anterior and the bottom is posterior. **(B) **Time-frequency and topographic maps of evoked power for HC and SZ, as in **A**. **(C) **Dipole model of the ASSR based on the HC grand average. Labels are for left hemisphere tangential (LH tan), left hemisphere radial (LH rad), right hemisphere tangential (RH tan), right hemisphere radial (RH rad), and Residual (Res) dipoles. **(D) **Global field power (blue) of the scalp ASSR and percent residual variance (%RV) of the dipole model (red). The period during which the dipole model was fit (30–550 ms) is shaded.

The scalp phase-locking factor (PLF) and evoked power data are shown in Figure 1A &1B. Both measures showed maxima at fronto-central and lateral posterior sites, in agreement with dipolar sources in the superior temporal plane. To be consistent with prior scalp EEG studies, we measured PLF and evoked power at fronto-central sites (see Methods). As expected, PLF and evoked power were significantly reduced in schizophrenia patients (SZ) compared to healthy controls (HC) (PLF: *F*[1,32] = 6.03, *p *< 0.05; evoked power: *F*[1,32] = 4.43, *p *< 0.05). Both measures also had larger values over the right hemisphere (RH) than the LH across both subject groups (PLF: *F*[1,32] = 6.58, *p *< 0.05; evoked power: *F*[1,32] = 8.37, *p *< 0.01), but the Group × Hemisphere interactions were not significant (PLF: *F*[1,32] = 0.154, *p *= 0.697; evoked power: *F*[1,32] = 0.007, *p *= 0.936).

### Source localization

Most studies in which source localization of the ASSR is performed have utilized magnetoencephalography (MEG), which is primarily sensitive to sources that are tangential to the scalp surface [28]. With MEG, the ASSR is well-fit by 2 dipoles, one each in the primary auditory cortex of the LH and RH that are oriented normally to the superior temporal plane (e.g., [10,26]). (Although these dipoles are radial to the frontal scalp surface, they are tangential to the lateral scalp which is closer, and hence are detectable by MEG.) The EEG is sensitive to both radial and tangential source orientations, and is more sensitive to deep sources than MEG. Not surprisingly, EEG studies have suggested that additional sources contribute to the ASSR besides the main superior temporal plane dipoles. These additional sources have been accounted for by dipoles in primary auditory cortex that are more radially-oriented than the tangential superior temporal dipoles, as well as a deep midline dipole that accounts for residual and/or subcortical activity [29,30].

ASSR source localization was performed with Brain Electric Source Analysis (BESA) v5.1 (see Fig 1C and 1D). BESA models brain activity as the contributions of dipolar sources with unique temporal patterns [31]. The goodness of fit of a dipole model is evaluated by its residual variance (RV) – the lower the RV, the better the fit. The HC grand average ASSR was filtered from 13–100 Hz to eliminate the transient auditory evoked potentials, and the 30–550 ms segment of the resulting waveform (the period of the ASSR) was used for dipole modelling with the standard BESA 4-shell (brain, scalp, skull, and cerebrospinal fluid) spherical head model. (It should be noted that the reported RV values are averaged over the period during which the dipole model is fit. Since the ASSR waveform is an oscillation, the RVs fluctuate between the peaks and zero-crossing points of the waveform, at which the RVs are best and worse, respectively [see Figure 1D]. Therefore the average RV reported by BESA is actually an underestimate when oscillatory activity is modeled.)

Our procedure for constructing the dipole model was similar to the "regional source" modeling procedure that has been utilized in numerous dipole modeling studies of auditory cortex activity (e.g., [29-31]). First, 2 dipoles were simultaneously fit with a symmetry constraint (that is, the dipoles were free to vary in orientation but were constrained to occupy homologous positions in each hemisphere). This 2-dipole model converged to deep midline positions. Since the 2-dipole model was unrealistic, a 4-dipole model was fit. This model consisted of 2 symmetrically-constrained dipoles as before, and 2 additional dipoles, each bound to the position of one dipole in the first pair but with unconstrained orientations. The dipoles in this model were simultaneously fit and converged to lateral temporal locations (8.9% RV). Finally, a fifth dipole was added to the 4-dipole model. This dipole converged to a deep midline position (6.7% RV). Thus, the final 5-dipole model consisted of 2 pairs of dipoles in the superior temporal plane of each hemisphere, and a deep midline dipole accounting for residual activity (Figure 1C). The locations and orientations of the dipoles are given in Table 1. This model is similar to those of Herdman et al. [29] and Poulsen et al. [30].

**Table 1:** Dipole locations (loc) and orientations (orient) for the source model derived from the HC data.

	X loc	Y loc	Z loc	X orient	Y orient	Z orient
LH tangential	-0.53	0.08	0.11	0.1	0.3	0.9

LH radial	-0.53	0.08	0.11	0.5	-0.8	-0.5

RH tangential	0.53	0.08	0.11	0.0	0.2	1.0

RH radial	0.53	0.08	0.11	-0.1	-1.0	-0.3

Residual	-0.07	-0.40	-0.01	0.0	-0.5	-0.9

Values are given in the BESA unit sphere coordinate system.

We adopted the following terminology for labelling the dipoles: The dipoles that are oriented tangentially to the lateral scalp surface (but radially to the fronto-central scalp) are here termed "LH tangential" and "RH tangential", and are the same dipoles as are typically found in MEG ASSR studies. The complementary cortical dipoles that have less tangential orientations are termed "LH radial" and "RH radial" (but it should be noted that their orientations are not strictly radial). The deep midline dipole is termed "Residual".

This modeling procedure was repeated numerous times with different initial conditions but always converged to the same solution. The procedure was also conducted without the symmetry constraint for the 4-dipole model, which resulted in a RV of 8.8%, but with the LH dipoles occupying a position that was unrealistically medial and deeper than with the symmetry constraint.

We also attempted to model the grand average ASSR of the SZ group but the results were unsatisfactory (the RV was 34.4%), likely due to the overall reduction in ASSR strength and the typical decrease of signal-to-noise ratio in the SZ data. Therefore, the HC model was used for both the HC and SZ data. When the 5-dipole HC model was applied to the SZ grand average ASSR the RV was 15.2%. It should be noted that while there are reductions in the size of portions of the brain in schizophrenia (including the left Heschl's gyrus; e.g., [32]) that might reduce the validity of using the same dipole model for HC and SZ, these reductions are not expected to be significant when compared with the anatomical accuracy of the present method. Furthermore, Teale et al. [33] reported that the hemispheric asymmetry in the anterior-posterior location of the ASSR "tangential" dipoles was reduced in schizophrenia. The asymmetry they found in their control subjects with MRI co-registered MEG was 6 mm, a difference which is likely to be undetectable with our methods.

### Source data

Finally, single-trial source waveforms were estimated for each subject. This was accomplished in the BESA source analysis module by selecting the HC dipole model and reading in the single trial data. BESA computes the projection of the single trial data into source space, estimating how the current scalp EEG data would be accounted for by the given dipole model. The single-trial source waveforms were then output from BESA for time-frequency analysis. PLF and evoked power were computed on the single-trial source waveforms as for the scalp electrode data.

**Figure 2:**
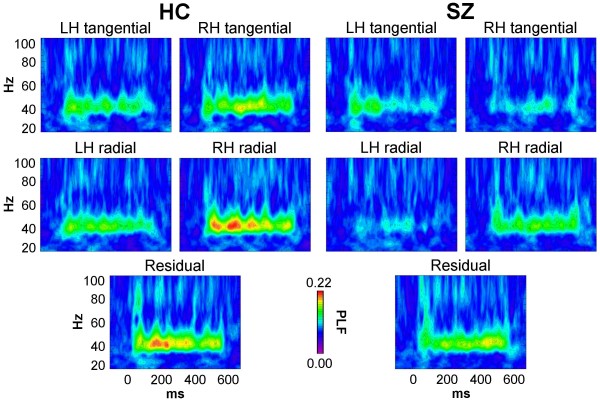
**PLF time-frequency maps of source activity for HC and SZ**.

The PLF source data are shown in Figure 2. PLF was reduced overall in SZ compared to HC (*F*[1,32] = 5.70, *p *< 0.05). There was a main effect of Source (*F*[4,128] = 6.07, *p *< 0.0001), indicating that the PLF of the sources differed, but the Group × Source interaction was not significant (*F*[4,128] = 0.938, *p *= 0.427). Post-hoc *t*-tests (Bonferroni-corrected) found that PLF of the RH tangential dipole was significantly reduced in SZ (*t*[32] = 2.80, *p *< 0.05), while the PLF reduction of the LH radial dipole in SZ was nearly significant (*t*[32] = 2.73, *p *= 0.050). (The rationale for the dipole labeling scheme is given below in the Methods.)

**Figure 3:**
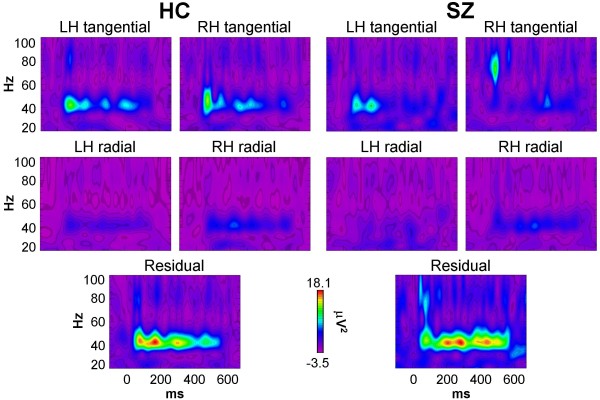
**Evoked power time-frequency maps of source activity for HC and SZ**.

The evoked power source data are shown in Figure 3. Evoked power tended to be reduced in SZ compared to HC, but in contrast to the scalp EEG data, the Group effect was not significant (*F*[1,32] = 0.313, *p *= 0.580), nor was the Group × Source interaction (*F*[4,128] = 0.402, *p *= 0.712). The main effect of Source was significant (*F*[4,128] = 6.02, *p *< 0.001). To explore the reason for the non-significant group difference in evoked power, *t*-tests were conducted for each source. Three of the sources showed non-significant reductions in SZ relative HC (RH tangential: *p *= 0.196; LH radial: *p *= 0.202; LH tangential: *p *= 0.688), while the other 2 sources had slightly increased evoked power in SZ (RH radial: *p *= 0.999; Residual: *p *= 0.859).

Evoked power and PLF are complementary measures of the same oscillatory activity. While the mathematical differences between these measures are clear (they both measure activity phase-locked to a reference event, but PLF is insensitive to power; see Methods), the extent to which they differ in practice is not well understood at this time. For instance, since PLF is independent of oscillation power, it may be more sensitive to signals with a poorer signal-to-noise ratio than evoked power. To examine the relationship between evoked power and PLF at the source level, we computed correlations between these measures for each cortical dipole. In HC each dipole showed strong positive correlations (RH tangential: ρ = 0.706, *p *< 0.01; RH radial: ρ = 0.608, *p *< 0.05; LH tangential: ρ = 0.761, *p *< 0.001; LH radial: ρ = 0.840, *p *< 0.0001). In contrast, SZ showed strong positive correlations for the RH dipoles while the LH dipole correlations were weaker, particularly for the LH radial dipole (RH tangential: ρ = 0.870, *p *< 0.0001; RH radial: ρ = 0.794, *p *< 0.0001; LH tangential: ρ = 0.445, *p *= 0.064; LH radial: ρ = 0.293, *p *= 0.238). These results may be interpreted as a decoupling of oscillation power and phase locking in the left auditory cortex in schizophrenia. On the other hand, the coupling between oscillation power and phase locking was preserved in schizophrenia in the right auditory cortex, despite overall reductions in these measures. As our understanding of the relationships between these measures grows, the meaning of these patterns will become clearer.

### Correlations between EEG measures and hallucination symptoms

**Figure 4:**
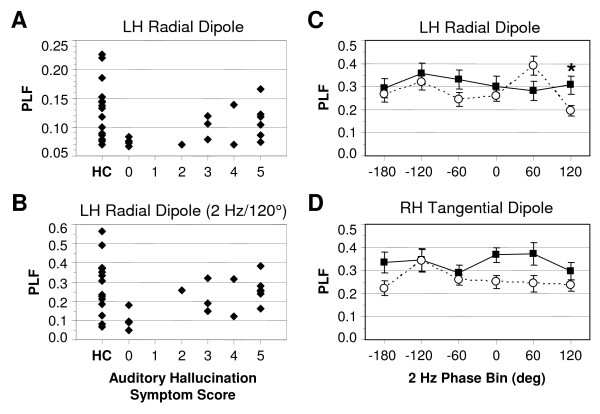
**Auditory hallucination correlations and cross-frequency interaction data**. **(A and B) **Scatterplots of LH radial dipole PLF vs. Auditory Hallucination symptom ratings. The HC values are displayed for comparison with the range of SZ values. **(A) **LH radial dipole PLF over all trials (ρ = 0.541, *p *< 0.05). **(B) **LH radial dipole PLF in the 120–180° phase bin for 2 Hz modulation (ρ = 0.617, *p *< 0.01). **(C and D) **ASSR PLF plotted as a function of 2 Hz phase for the LH radial **(C) **and RH tangential **(D) **dipoles. HC = filled squares, SZ = open circles. In **(C)**, the asterisk indicates the significant reduction of LH radial dipole PLF in SZ for the 120–180° phase bin.

To test our hypothesis that oscillatory activity in the left primary auditory cortex would be positively correlated with auditory hallucination symptoms, we computed correlations between source PLF and evoked power and the Auditory Hallucination item of the Scale for the Assessment of Positive Symptoms (SAPS) [34]. A significant positive correlation was found for PLF of the LH radial dipole (ρ = 0.541, *p *< 0.05, N = 17) (Figure 4A). There were no significant correlations for PLF of any of the other sources (ρ's < |0.295|, *p*'s > 0.250; N = 17). For evoked power there was a similar pattern of results: a significant positive correlation for the LH radial dipole (ρ = 0.486, *p *< 0.05, N = 17), but no significant correlations for the other sources (ρ's < |0.229|, *p*'s > 0.376, N = 17). None of the other source measures were correlated with any of the other SAPS hallucination items (Voices Commenting, Voices Conversing, Somatic/Tactile, Olfactory, Visual) (ρ's < |0.391|, *p*'s > 0.121, N = 17). Furthermore, no correlations were found between the Auditory Hallucinations item and scalp PLF (ρ = 0.143, *p *= 0.585, N = 17) or evoked power (ρ = 0.060, *p *= 0.820, N = 17) measures at the fronto-central electrodes, where the ASSR deficit in SZ was observed.

### Cross-frequency interactions

Lastly we conducted exploratory analyses to examine whether the ASSR PLF deficit in SZ was modulated by the phase of oscillations in the δ (1–4 Hz) and/or θ (4–8 Hz) frequency bands (see Methods). Cross-frequency interactions were tested at the frequencies of 2, 4, 6, and 8 Hz for the RH tangential and LH radial dipole data. A significant Group × Phase Bin interaction (*F*[5,160] = 3.46, *p *< 0.05) was observed only for the LH radial dipole data at 2 Hz (Figure 4C). Follow-up analyses found a main effect of Phase Bin for SZ (*F*[5,85] = 6.54, *p *< 0.001) but not HC (*F*[5,75] = 0.717, *p *= 0.576). Exploratory *t*-tests indicated that PLF for this source was significantly decreased in SZ relative to HC in the 120–180° phase bin (*t*[32] = 2.49, *p *< 0.05). In this bin, LH radial dipole PLF in SZ was positively correlated with the Auditory Hallucination item of the SAPS (ρ = 0.617, *p *< 0.01) (Figure 4B). PLF in the 0–60° phase bin was also positively correlated with the Auditory Hallucinations item (ρ = 0.572, *p *< 0.05). ASSR evoked power at 2 Hz did not differ between HC and SZ.

### Correlations with demographic and clinical variables

There were no significant correlations between scalp and source EEG effects and the demographic and clinical variables listed in Table 2.

**Table 2:** Comparison of demographic and clinical variables for the HC and SZ groups.

	HC	SZ	Statistic	*p*
Age (years)	44.4 +/- 6.8	39.8 +/- 10.5	*t*[32] = 1.47	0.151

Parental socio-economic status (ref. 24)	3.1 +/- 1.3	3.2 +/- 0.9	*t*[28] = -0.316	0.754

Handedness	0.78 +/- 0.16	0.77 +/- 0.25	*t*[32] = 0.172	0.864

Age of onset (years)		26.1 +/- 8.0		

Positive symptom total (SAPS)		9.0 +/- 4.2		

Negative symptom total (SANS)		13.9 +/- 4.9		

Medication dosage (chlorpromazine equivalents)		450 +/- 306 range 83–1200		

Mean +/- standard deviation are given for each variable.

## Discussion

Based on previous findings of positive correlations between high-frequency oscillatory activity and hallucination symptoms in schizophrenia patients, we hypothesized that the left auditory cortex response to 40 Hz auditory steady-state stimulation would be correlated with auditory hallucinations in schizophrenia patients. Phase locking and evoked power of the 40 Hz ASSR were decreased in SZ compared to HC, as expected. However, the phase locking and evoked power of a LH ASSR source were positively correlated with the Auditory Hallucinations symptom rating of the SAPS. Thus, patients with higher γ activity in the left primary auditory cortex had a greater propensity for experiencing auditory hallucinations. Furthermore, we found evidence that the degree of phase locking of this LH source was modulated by oscillatory activity at 2 Hz in SZ but not HC.

Three main patterns of abnormalities were observed at the source level in the present study: 1) PLF was reduced in the SZ group for the RH tangential and LH radial dipoles (the latter being a weaker effect). 2) LH radial dipole PLF was positively correlated with auditory hallucination symptoms (which may have offset the overall reduction of LH radial dipole PLF in the SZ group). 3) The coupling between evoked power and PLF was reduced in the SZ group for the LH but not the RH sources. The paradoxical finding of a positive correlation between auditory hallucination symptoms and LH radial dipole PLF, in the context of overall reductions of RH tangential and LH radial dipole PLF, suggests that the left and right auditory cortices may be differentially impaired in schizophrenia.

One possible explanation for the different impairments is that there is a single circuit abnormality underlying both patterns which causes the reduction in ASSR PLF, and this abnormality is expressed in different ways in the left and right auditory cortex. As a reviewer of this manuscript proposed, the ability to downregulate the ASSR could be an adaptive response to neural circuit dysfunction in schizophrenia. This adaptive response could be impaired in the left auditory cortex of patients who experience auditory hallucinations despite receiving antipsychotic treatment, so that the LH ASSR is "preserved" in these patients. This hypothesis takes into account the potential effects of adaptive homeostatic responses, which are likely to play a critical role in shaping the functional consequences of neural circuit abnormalities in schizophrenia (e.g., [35]).

Another possibility is that at least two distinct neural circuit abnormalities may be present in the auditory cortex in schizophrenia. The first abnormality is responsible for the overall 40 Hz ASSR deficit, and is most associated with the RH tangential dipole. This finding is consistent with previous studies that have shown that the right auditory cortex is the predominant generator of the ASSR [e.g., [9,10]]. One possible cause of such a deficit is reduced synaptic connectivity [36]. Sweet et al. [21,22] found reductions of pyramidal cell somal volume (which is correlated with the extent of axonal and dendritic arborization; e.g., [37]) and axon terminal density in deep layer 3 of primary auditory cortex, the likely generator of the ASSR. Consistent with the microscopic evidence of reduced synaptic connectivity, reductions in the volume of Heschl's gyrus in schizophrenia patients have been reported in magnetic resonance imaging (MRI) studies (reviewed in [38]). There is also evidence that these reductions, which are apparent in first-episode schizophrenia (e.g., [39]), progress over time in the left hemisphere [32,40]. Furthermore, the surface area of Heschl's gyrus was found to be reduced in a study of post-mortem samples [38].

The second circuit abnormality implied by the present results is manifested as an increase in γ activity in the left auditory cortex in association with an increased propensity for auditory hallucinations. This abnormality is associated with the LH radial dipole. If the neural substrate of a perceived object is an attractor state in the brain, such as oscillatory synchronization among cells coding individual features of the object [1], then hallucinations could result from an increased propensity for a dysfunctional network to enter attractor states independently of external biasing influences [2,8,41,42]. Possible external biasing inputs include sensory stimulation or an attentional bias signal from prefrontal cortex [43]. The dysfunctional network would consist of sensory areas and possibly association cortex, depending on the complexity of features making up the hallucination. Delusions might originate from a similar dysfunction in higher-level areas. Olypher et al. [44] have reported evidence supporting this proposal in electrophysiological recordings from the rat hippocampus.

As noted above, we have found positive correlations between hallucination symptom ratings and the phase locking of γ and β oscillations in previous studies [2,3], and Baldeweg et al. [11] and Ropohl et al. [12] have reported evidence for increased γ activity during hallucinations in case studies. Evidence from other sources points to an association between increased intrinsic levels of cortical activity and hallucinations. In a functional MRI study of patients with Charles Bonnet syndrome, ffytche et al. [45] reported that the visual cortex of visual hallucinators had an increased level of intrinsic baseline activity. Hoffmann and colleagues have found that the application of slow repetitive transcranial magnetic stimulation (rTMS) to the left posterior temporal-parietal cortex alleviated auditory hallucinations in schizophrenia patients with medication-resistant hallucinations (e.g., [46]). Since slow rTMS decreases cortical excitability, the findings of Hoffman et al. imply that the language-related association cortex in auditory hallucinators is in a hyperexcited state [47]. Similarly, Merabet et al. [48] reported that slow rTMS applied to the occipital lobe reduced visual hallucinations in a case study. More general evidence for cortical hyperexcitability in schizophrenia has been found, not just related to hallucinations. For instance, Heckers et al. [49] detected increased baseline activity in the hippocampus with positron emission tomography, and TMS studies have documented a range of deficits of cortical inhibition (reviewed in [50]).

Cortical hyperexcitability can be produced by the antagonism of N-methyl-D-aspartate (NMDA) receptors (e.g., [51,52]), which also results in a constellation of positive and negative symptoms and cognitive deficits in healthy individuals that closely resembles aspects of schizophrenia (e.g., [53,54]). Recent studies with animal models have demonstrated that NMDA receptor antagonists can increase or decrease the power of γ oscillations, depending on the brain region [55,56]. These effects of NMDA receptor antagonism on γ activity are probably mediated mainly through parvalbumin-expressing, fast-spiking inhibitory interneurons, which are crucial elements of the circuitry that generates γ oscillations [57]. The abnormalities of these interneurons found in post-mortem studies of schizophrenia patients can be produced by NMDA receptor antagonists (reviewed in [35,58]). Therefore, the evidence suggests that hallucinations in schizophrenia might result, at least in part, from aberrant oscillatory synchronization associated with cortical excitability and NMDA receptor hypofunction in sensory cortex. The positive correlations between hallucination symptoms and β/γ activity we have observed may be due to NMDA receptor hypofunction in sensory areas. Further experiments are necessary to test this hypothesis.

Lastly, the decoupling of evoked power and PLF for the LH but not the RH sources in the SZ group is an additional piece of evidence which suggests that different abnormalities may be present in the left and right auditory cortices. This finding could support either of the two hypotheses presented above.

In analyses of cross-frequency interactions, we found that the LH radial dipole ASSR in schizophrenia patients was modulated by oscillatory activity at 2 Hz, in the δ band. ASSR PLF was significantly reduced in patients compared to controls for phases of 2 Hz activity between 120–180°, and was positively correlated with auditory hallucinations. (In fact, the magnitude of the correlation was greater than in the non-phase modulated data.) Recently there has been much interest in interactions between oscillations at different frequencies, with studies demonstrating hierarchical relationships in which high-frequency oscillations can be modulated by the phase of low-frequency oscillations (reviewed in [59]). In recordings from the auditory cortex of awake macaques, Lakatos et al. [27] found that δ band phase modulated spontaneous θ activity, and θ phase modulated spontaneous γ activity. Here the δ phase modulation of the γ ASSR was abnormal, as it was only present in the schizophrenia patients and not the healthy controls.

That the δ modulation of the 40 Hz ASSR was found in a pathological state, rather than a normal state, may seem paradoxical. However, abnormal δ-band activity has been associated with cortical lesions [60], epileptogenic cortex [61], and NMDA receptor antagonism [62], so it is plausible that some type of cortical abnormality could influence δ activity in schizophrenia. In fact, studies of resting state EEG have demonstrated enhanced δ-band power in schizophrenia [63,64]. In the present study the power of the 2 Hz oscillation tended to be reduced in patients relative to controls, but this was measured during the ASSR. In future research we will examine whether resting-state δ activity is associated with the pathological δ modulation of the 40 Hz ASSR.

It is important to note that our hypothesis does not exclude the possibility that hallucinations result from abnormalities in other neural systems. Functional neuroimaging studies have documented that a variety of cortical areas are involved in hallucinations (e.g., [14,16-18]). One possibility is that aberrant oscillatory synchrony in sensory cortex could interact with dysfunctional efference copy mechanisms (reviewed in [65]). Also, it should be kept in mind that here we have reported correlations with hallucination symptom ratings that measure the lifetime history of hallucinations in the patients. The schizophrenia patients in this sample were chronic and medicated, and were not actively experiencing hallucinations at the time of the study. (Due to ethical reasons the patients could not be taken off of their antipsychotic medication, and patients with treatment-resistant hallucinations are rare.) Presumably, actively hallucinating patients would show large-amplitude β/γ oscillations in the sensory cortex corresponding to the modality of their hallucinations, as suggested by the case reports [11,12]. Hence, it may be considered notable that the present findings were obtained in non-actively hallucinating patients. The positive correlations we have observed between hallucination symptoms and oscillatory measures may reflect a subthreshold level of dysfunction in the cortex that would produce hallucinations in the absence of antipsychotic medication.

An alternative hypothesis should be noted. Hong et al. [24] reported that the power of the scalp-recorded 40 Hz ASSR was larger in chronic schizophrenia patients who were taking atypical compared to typical antipsychotics. If the patients in the present study who had higher auditory hallucination symptom ratings also were taking higher doses of atypical antipsychotics, the positive correlation between auditory hallucination symptoms and LH PLF might be explained as a medication effect. However, this hypothesis does not seem likely for several reasons. First, we did not find a significant correlation between medication dosage and the Auditory Hallucinations ratings (ρ = 0.137, *p *= 0.600). Second, no effect of medication dosage was found on 40 Hz ASSR evoked power measured at the scalp level (the measure which Hong et al. found to be sensitive to medication type), nor on any of the dipole measures. Third, 7 of the 13 patients taking atypical antipsychotics in Hong et al. were on clozapine, which has been found to stimulate NMDA receptors [66] and to reverse some of the effects of NMDA receptor antagonism [67]. Therefore, the increase of 40 Hz ASSR power in the patients on atypical antipsychotics could have been due to half of them being on clozapine. However, only one of the patients in the present study was on clozapine, and this patient did not report any hallucination symptoms. Finally, Light et al. [25] did not find any differences in the 40 Hz ASSR between schizophrenia patients on typical compared to atypical antipsychotics in a larger sample than the Hong et al. study, so a definitive effect of medication type has yet to be established. But since the determination of chlorpromazine equivalency for atypical antipsychotics is inexact, medication effects in the present study cannot be ruled out.

No alteration of the hemispheric asymmetry of the ASSR deficit was found here. In a previous study we found a larger reduction of the 40 Hz ASSR at LH electrodes than RH electrodes in first-episode schizophrenia patients [3], so the absence of an asymmetrical ASSR deficit in the present study suggests that some aspect of chronicity (e.g., progression of the disorder, or long-term medication) could have normalized the deficit.

The use of source localization methods to measure the ASSR permits the testing of hypotheses with greater anatomical specificity than scalp EEG recordings, but the validity of the results rests upon the validity of the localization method. As a number of studies employing different localization methods have consistently localized the cortical sources of the ASSR to primary auditory cortex with MEG (e.g., [9,10,26]), and since the dipole model we used is very similar to other published models based on EEG [29,30], we are confident in the validity of our results. However, we note that one discrepancy in the present results was that source evoked power was not significantly decreased in SZ compared to HC, while this effect was observed in the scalp voltage data. On the other hand, the patients' PLF deficit in the scalp voltage data was reflected in the source PLF results. The reasons for this discrepancy are not clear. Using MEG, Teale et al. [26] found PLF and evoked power reductions of the tangential ASSR sources in both hemispheres in chronic schizophrenia patients. (However, differences in auditory stimulation could have led to different results, as Teale et al. used monaural stimulation, while the present study used binaural stimulation.) We also compared ASSR evoked power between groups using the HC model for the HC group and a similar 5-dipole model derived from the SZ grand average data for the SZ group (see Methods), but the overall group difference in evoked power still did not approach statistical significance (*p *= 0.621 compared to *p *= 0.253 for the HC model). A higher degree of anatomical specificity could be obtained with improvements in the source localization method, such as performing localization on the individual subjects' data using head models derived from their anatomical MRI scans.

## Conclusion

Two distinct neural circuit abnormalities may be present in the auditory cortex in schizophrenia. One is responsible for the overall 40 Hz ASSR deficit, and the other is manifested as an increase in phase synchronization of left auditory cortex activity, which is associated with an increased propensity for auditory hallucinations. The latter finding is consistent with prior reports of associations between β/γ oscillations and hallucinations. The left auditory cortex ASSR deficit and correlation with auditory hallucination symptoms may also be related to pathological cross-frequency interactions.

## Methods

### Subjects

This study was approved by the Institutional Review Boards of the Veterans Affairs Boston Healthcare System and Harvard Medical School. After a complete description of the study to the subjects, written informed consent was obtained. All subjects were paid for their participation.

Nineteen chronic SZ and 20 HC participated in the study. SZ were diagnosed according to DSM-IV criteria. Subjects were selected without regard for ethnicity, and met our standard inclusion criteria: 1) age between 18–55 years; 2) right-handed (so that possible hemispheric lateralization effects would not be obscured by left-handers with reduced or reversed functional laterality); 3) no history of electroconvulsive treatment; 4) no history of neurological illness, including epilepsy; 5) no history of alcohol or drug dependence, nor abuse within the last year, nor long duration (>1 year) of past abuse (DSM-IV criteria); 6) no present medication for medical disorders that would have deleterious EEG, neurological, or cognitive functioning consequences; 7) verbal IQ above 75; 8) no alcohol use in the 24 hours prior to testing; and 9) English as a first language.

The data from 1 SZ and 4 HC were excluded due to technical problems (1 HC), bad channels (1 SZ, 2 HC), or excessive artifacts (1 HC), resulting in a final sample of 18 SZ and 16 HC. Demographic and clinical data are presented in Table 2. Schizophrenic symptoms were assessed using the SAPS [34] and the Scale for the Assessment of Negative Symptoms (SANS; [68]). The final SZ and HC groups did not differ in gender (all male), age, parental socio-economic status [69], nor handedness (all right-handed) [70]. The diagnostic composition of the SZ group was 10 paranoid, 5 undifferentiated, 2 schizoaffective, and 1 disorganized. All of the final sample of patients were taking atypical antipsychotics at the time of the experiment. One patient was also receiving a typical antipsychotic. Antipsychotic medication dosages were converted to chlorpromazine equivalents [71].

### Hallucination symptom ratings

The hallucination symptom items on the SAPS were used as measures of the patients' lifetime hallucination history without reference to their hallucination status at the time of testing. We did not rate the patients' hallucination status at the time of the experiment because the patients in this study were stable, chronic outpatients, whose symptom profile generally does not change much over time. For example, a patient who has a moderate number of hallucinations might or might not have hallucinated recently at the time of test. If this patient's hallucination symptoms were rated at the present or very recent past, she/he might present at either extreme of the scale. Thus, a rating of symptoms focused on the present would fail to capture the true severity of the symptoms, and would have greater variance than a lifetime history.

### Stimuli and experimental design

Subjects were seated in a quiet room in a comfortable chair in front of a computer monitor. Stimuli consisted of 150 40 Hz trains of 1 ms white noise clicks (500 ms duration, 1100 ms stimulus onset asynchrony), and were presented binaurally through headphones (70 dB sound pressure level). Subjects were instructed to look at the fixation cross on the monitor and listen to the stimuli.

### Electrophysiological recording and processing

The EEG was recorded (0.05–100 Hz, 500 Hz digitization) with a Neuroscan Synamp amplifier using sintered Ag/Ag-Cl electrodes in an electrode cap at 60 standard scalp sites [72], nosetip, and left earlobe, referenced to the right earlobe, and grounded at AFz. Bipolar vertical and horizontal electro-oculograms were recorded from electrodes above and below the right eye and at the left and right outer canthi, respectively. Electrode impedances were <10 kΩ. Single-trial epochs were extracted from -250 to +772 ms relative to stimulus onset and corrected for ocular artifacts (blinks and eye movements) with independent component analysis [73]. Next, epochs containing other artifacts were removed. The artifact criteria were: 1) > +/- 90 μV change in one time point; and 2) amplitude range within an epoch exceeding 200 μV. These criteria were visually tested and verified. Finally, the retained artifact-free single epochs were re-referenced to the average reference. The number of epochs retained per subject was (mean +/- standard deviation) 145 +/- 7 for HC and 140 +/- 12 for SZ.

Event-related potentials and spectral measures were computed from the single-trial epochs. Time-frequency decomposition was performed using the Morlet wavelet transform [74], which was applied in 1 Hz steps from 1–100 Hz at each time point to yield time-frequency maps. The wavelet frequency/duration ratio f_0_/σ_f _was 6. Two event-related spectral measures were computed: PLF and evoked power. PLF measures the variance of phase across single trials, and is independent of power. PLF is computed as one minus the circular variance of phases and ranges from 0 (random distribution) to 1 (perfect phase locking). Evoked power measures the power of the average evoked potential in which the contribution of non-stimulus locked activity is minimized. Average baseline values in the pre-stimulus period from -100 to 0 ms were subtracted from the evoked power time-frequency maps. No baseline correction was performed on the PLF maps, as there was minimal variance in this measure during the pre-stimulus period.

### Cross-frequency interaction analysis

Cross-frequency interaction was investigated using a method similar to that of Lakatos et al. [27]. The set of single trial epochs for each subject and ASSR source were decomposed in the frequency domain with a Fast Fourier Transform (FFT). The FFT was applied to the 50–550 ms range of the epoch (spanning the ASSR) and Hanning windowed. The resulting frequency resolution was 2 Hz. The phase of the EEG on each single trial at frequencies in the δ and θ bands (2, 4, 6, and 8 Hz) and at 40 Hz was calculated from the FFT data. The single trials were sorted according to their phase at each of the specified δ/θ frequencies into bins of 60° (-180 to -120°, -120 to -60°, -60 to 0°, 0 to 60°, 60 to 120°, and 120 to 180°). PLF was calculated on the 40 Hz phases in each low-frequency phase bin. This procedure resulted in a data set for each subject consisting of 40 Hz PLF values for each low-frequency phase bin and ASSR source.

### Statistical analysis

In the scalp EEG and source data, average PLF and evoked power were computed within the time-frequency window of 30–550 ms and 38–50 Hz where the ASSR was maximal. Each measure was averaged across all time points and frequencies within this window. In the scalp EEG data, PLF and evoked power were measured across the fronto-central electrodes where the ASSR was maximal for these two measures: F1/2, F3/4, FC1/2, and FC3/4. These measures were analyzed in ANOVAs with the design Group (HC/SZ) × Hemisphere (LH/RH) × Midline Site (Frontal/Fronto-Central) × Lateral Site (1/2, 3/4). Source PLF and evoked power measures were analyzed in ANOVAs with the design Group (HC/SZ) × Source (LH tangential/RH tangential/LH radial/RH radial/Residual). Cross-frequency interaction data were analyzed in ANOVAs with the design Group (HC/SZ) × Phase Bin (-180 to -120, -120 to -60, -60 to 0, 0 to 60, 60 to 120, and 120 to 180°).

The Greenhouse-Geisser correction for inhomogeneity of variance [75] was applied for factors with more than two levels and is reflected in the reported *p *values. Since the PLF measure is bounded between 0 and 1 and may not be normally distributed, PLF effects detected in ANOVAs were confirmed with non-parametric statistics (not reported here). The non-parametric Spearman's ρ was used for correlation analyses (two-tailed). All post-hoc statistical tests were Bonferroni-corrected for multiple comparisons except where noted.

## Authors' contributions

KMS designed and conducted the study, analyzed the data, and wrote the paper. RWM, PGN, MAN, and MES also contributed to writing the paper. All authors read and approved the final manuscript.
